# Tattoo Removal in Forensic Mental Health Settings: A Case for Advocacy

**DOI:** 10.7759/cureus.94997

**Published:** 2025-10-20

**Authors:** Nandini Mishra, Priya Khalsa, Achal Mishra

**Affiliations:** 1 Medicine, Glasgow Royal Infirmary, Glasgow, GBR; 2 Psychiatry, University of Toronto, Toronto, CAN; 3 Forensic Psychiatry, Waypoint Centre for Mental Health Care, Penetanguishene, CAN

**Keywords:** advocacy, mental health, self-esteem, stigma, tattoo removal

## Abstract

The significance of visible tattoos in psychiatric patients should be explored with them. This can reveal important information about their psychological well-being. Facial tattoos can impact the individual’s appearance and self-esteem, especially if the tattoo(s) were acquired during an episode of mental illness. It serves as a constant reminder of the time when the person was unwell and can hinder progress and rehabilitation. Removal of such tattoo(s) can aid in the recovery of these patients. While tattoo removal services are available, patients with chronic and severe mental health problems often lack the funds for tattoo removal. Similarly, patients within forensic mental health settings may have additional restrictions on their movement, which serve as a barrier to accessing treatment. We would like to discuss the case of a young male patient who is undergoing tattoo removal with significant improvement in self-esteem and call for advocacy in this regard.

## Introduction

Tattoos serve varying purposes for different cultures and people across the world. They can be a means of self-expression, affiliation with a group, self-identity, or religious or spiritual in nature [1, 2]. The earliest evidence of tattoos dates back to around 4000-3000 BC, from naturally mummified bodies of males and females found in Egypt [3]. Tattooing has been practiced across the globe, from Oceania, Asia, Africa, and the Americas, and can represent beauty, art, and fashion, social status, a rite of passage, and cultural traditions [4].

Tattoos within correctional/prison settings can indicate not only gang affiliation but also hierarchy and career status within the gang [5]. Recognizing tattoos within correctional and forensic mental health settings is important in the rehabilitation of subjects and may indicate their level of dangerousness and risk of recidivism [6].

There is some evidence to suggest that the number of tattoos and body piercings was related to the emotional distress and high-risk behaviors displayed by the individual [2]. Tattoo regret is not uncommon, with one in five people in the USA reporting this [7]. There is emerging evidence to suggest that tattooing may have a positive effect on the mental health of the individual and aid in recovery, along with enabling positive body image and empowerment [8].

We present the case of a patient with multiple visible tattoos (including facial) that caused the individual significant distress and the positive impact of their removal on the person's psychosocial functioning.

## Case presentation

**Figure 1:**
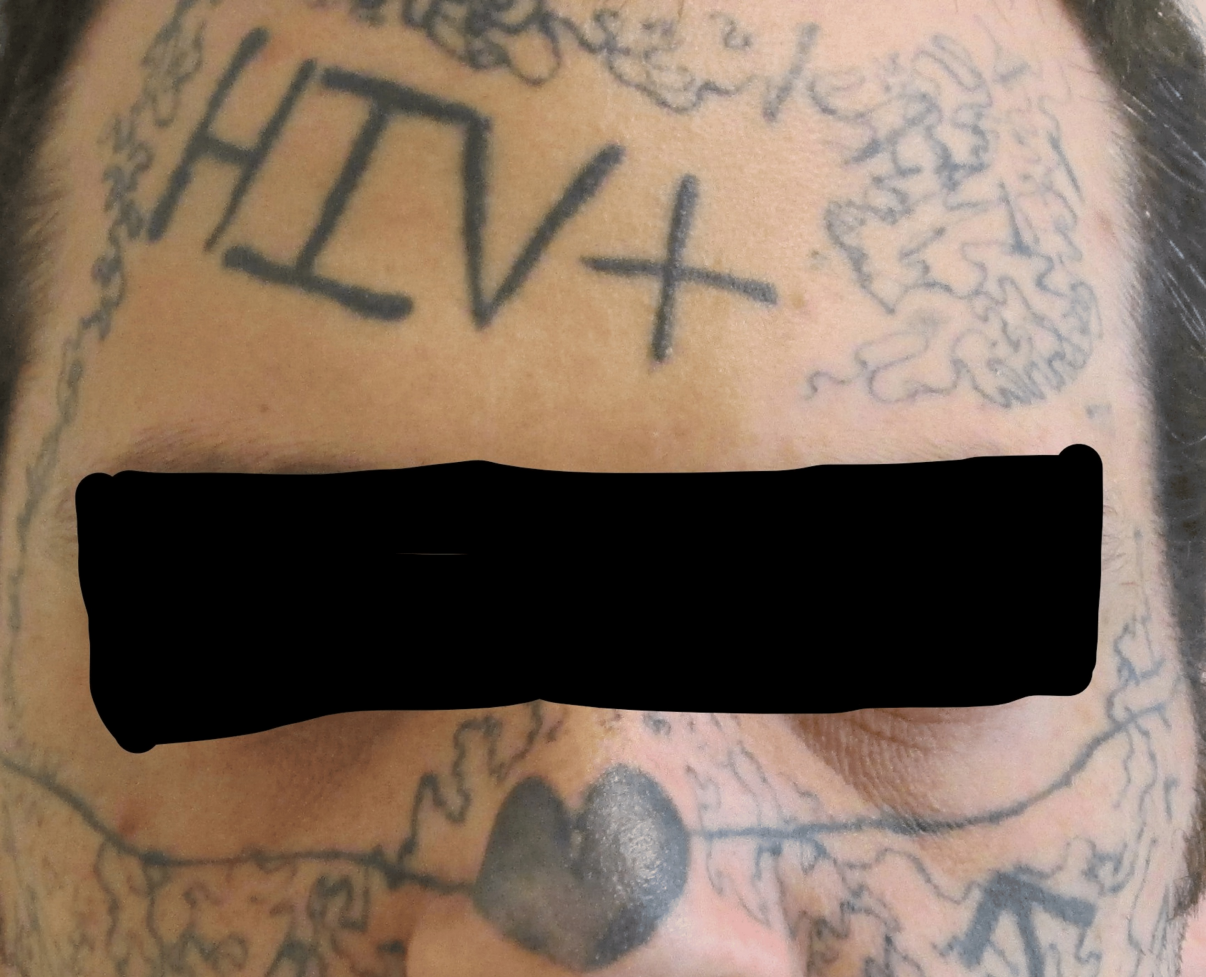
Photograph of the patient prior to starting treatment highlighting the prominence of the facial tattoos

The patient was a 35-year-old male who was an inpatient at a forensic psychiatric hospital in Ontario, Canada, detained on the offenses of criminal harassment and uttering threats to cause death. He was diagnosed with unspecified schizophrenia spectrum and other psychotic disorders, attention deficit hyperactivity disorder, and antisocial personality disorder. He had acquired multiple tattoos on various parts of his body over many years, starting at the age of 17. Some of these were acquired from professionals, others were done by himself and friends. Many of these tattoos, including the facial ones, were acquired while in jail. The location of these tattoos included the face, neck, chest, hands, and arms. Many of the tattoos, especially on the face (nose, cheeks), neck, and hands, were prominent and visible, as visible in the photograph. The patient has given written informed consent to display the photographs (Figure 1). 

The facial tattoos had caused the patient considerable distress over the years. These were acquired when he was experiencing an episode of psychosis during which his thinking was influenced by delusional beliefs. At the time he was studying philosophy and was preoccupied with "the original sin...equating it with existential circumstances...no such thing as true love, everything was an illusion, had a relationship break-up, did not want to be with anyone, love was a lie, wanted to be unattractive, put HIV-positive (not correct) to put people off." He said that the other tattoos were acquired for various reasons, including for an intimate partner, personal desire, and to be part of the neighborhood or a group, saying, “(They are) the only roadmap of identity I have ever had.” 

The facial tattoos in particular attracted unwanted attention from others due to their location, especially the nose. He had a tattoo with the name of the victim of one of his offenses on his face that served as a constant reminder of the past. These tattoos had been a significant barrier to pro-social and recreational activity. He had once in the community been denied the opportunity to join a gym because of these tattoos. He was worried about the social repercussions of the tattoos, as people had bullied him and made rude and derogatory comments. Because of the tattoos, he felt that people constantly judged him negatively. He was especially concerned about the social impact of the tattoos, stating, “Since the face tattoos, I worry about potential for external consequences and the future; people make comments-rude, derogatory, and judgmental comments.”

Another motivation for having the tattoos removed was to be able to have an intimate relationship when the opportunity arose. He was worried that, due to the nature of the tattoos, as visible in the photograph, finding a partner would prove especially challenging. He wanted to have tattoo removal to transition to a healthier lifestyle and gain social acceptance, summarizing the challenges he encountered due to the facial tattoos as “social alienation, problems with jobs, and no one will enter into a stable relationship with someone who has an HIV+ tattoo on their forehead.”

Treatment

The patient’s ongoing treatment included a long-acting monthly injection of the antipsychotic medication aripiprazole (Abilify Maintenna 400 mg IM every 28 days) and the stimulant medication lisdexamfetamine (Vyvanse 50 mg daily orally). He had been on this regimen for over 12 months each, and his psychosis was in remission. He attended various recreational programs in the hospital, including the recreational center, which houses a fitness center. He had declined any talk therapy for his concerns about appearance, but was willing to consider it after the tattoo removal treatment was complete.

The cost of tattoo removal in Ontario, Canada, can be prohibitive. An average session costs upwards of $400. This was very unaffordable for an individual who was supported by social benefits and received only a small amount of money (around $150 per month) due to being in the hospital. The number of sessions required for the removal of these tattoos would be multiple, along with the need to travel.

A clinic offering tattoo removal was located. Due to the nature of the tattoos and their location and visibility, upon request, the clinic agreed to offer tattoo removal free of charge. The hospital arranged back-and-forth transport along with a staff escort and paid for this. At the time of writing this article, the patient had received 15 treatments. These alternated between different sites. 

The tattoo removal clinic undertook comprehensive pretreatment counselling and imparted information about the risks and benefits of the treatment, including the aftercare, prior to treatment. The patient underwent an assessment of the areas to be treated to exclude the presence of any underlying lesions. The risks of complications, including the challenges with diagnosing melanoma following tattoo removal, were discussed with the patient by the dermatological team. The aftercare instructions were shared with the inpatient nursing team to allow them to assist the patient with aftercare and monitor the patient for possible complications of treatment, which can include blistering, bruising, tenderness, itching, and swelling.

**Figure 2:**
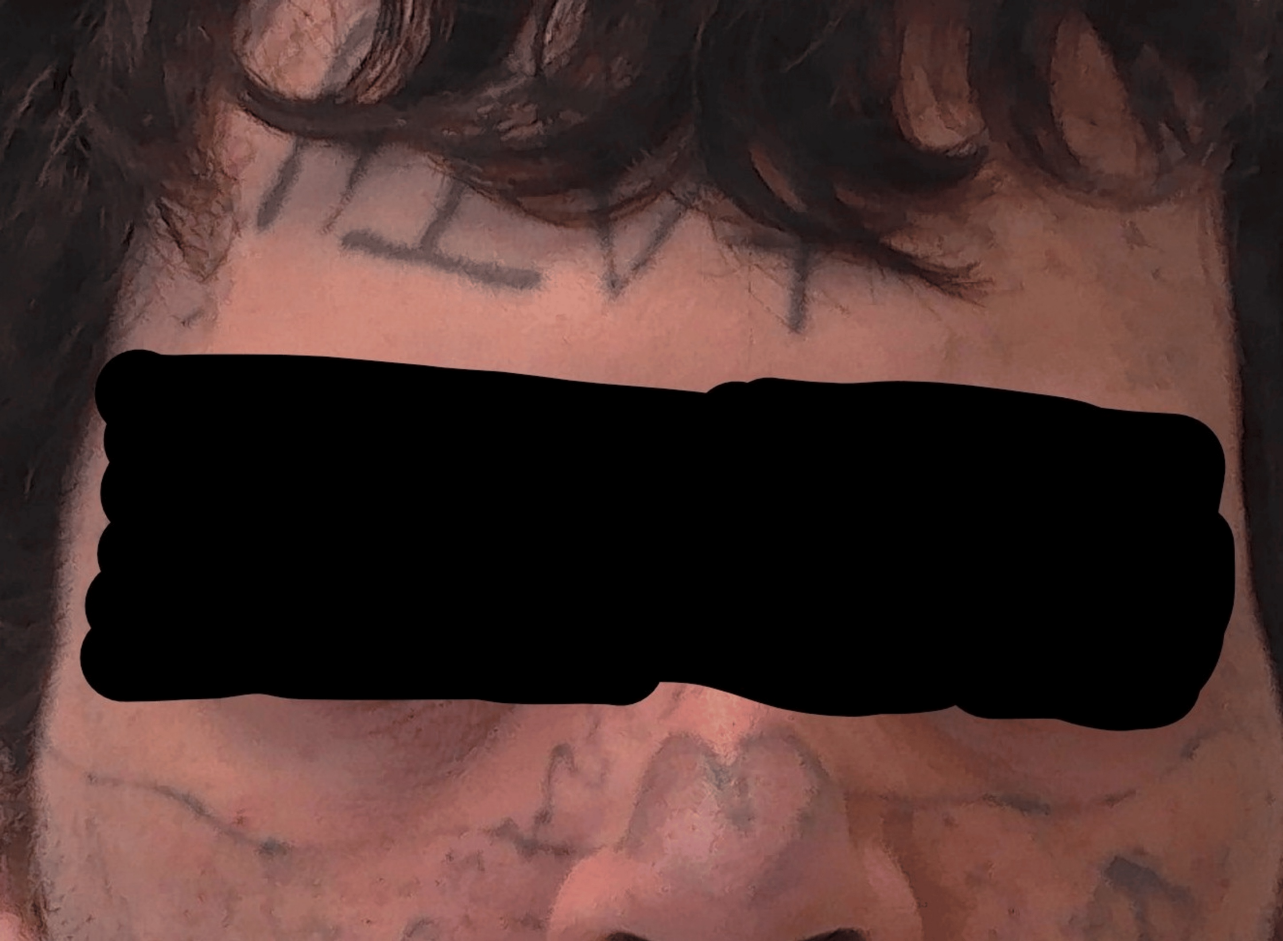
Photograph following treatment (ongoing) highlighting the reduction in the prominence of the facial tattoos.

The patient reported a significant improvement in his self-confidence following treatment. Figure 2 shows evidence of the change in his appearance. He was especially pleased with the reduction in the prominence of the tattoos on his nose and face.

We could not find any questionnaire that fit our purpose of assessing the psychological distress and social challenges related to tattoos for this particular patient. We used two questions to rate the emotional distress and social challenges faced by the patient related to the tattoos. The patient answered both questions as 5, or severe, prior to treatment, indicating extreme distress and social challenges.

The questions were: 1. How would you rate your emotional distress and preoccupation with the tattoos, on a scale of 1 to 5? (1= no distress, 3= moderate, and 5= severe). 2. How would you rate the problems that you have faced in social and leisure functioning due to the tattoos on a scale of 1 to 5? (1= none, 3= moderate, and 5= severe).

Following treatment (ongoing), the patient reported a considerable improvement in how he felt about himself. He said, “My self-esteem is better; I like my face.” He was happy with the outcome, and when asked about his social interactions, he said, “I feel more confident with people; I can see hope now.” He denied any side effects from the treatment. He rated the response to both the above questions at 3, or moderate, following treatment that is ongoing. He reported a significant reduction in the constant preoccupation with his appearance. It allowed him to wear a cap or keep his hair over his forehead to cover the HIV+ tattoo for the time being, until it could be treated.

## Discussion

Tattoos serve various purposes for different groups of people across the world. They can be a means of self-expression, affiliation with a group, self-identity, or religious or spiritual in nature [1, 2].

Tattoos within correctional/prison settings can indicate not only gang affiliation but also hierarchy and career status within the gang [5]. Recognizing tattoos within correctional and forensic mental health settings is important in the rehabilitation of subjects and may indicate their level of dangerousness and risk of recidivism [6].

Tattoo regret is common, with one in five people in the USA reporting this. The stigma relates to people with tattoos being more likely to be perceived as less attractive, intelligent, and professional, and more rebellious. Having a visible tattoo on the face, neck, hands, wrists, or fingers; getting a tattoo because of peer pressure; being impaired when getting a tattoo; and experiencing an adverse event related to a tattoo were predictive of tattoo regret [7]. There is some evidence to suggest that the number of tattoos and body piercings was related to the emotional distress and high-risk behaviors displayed by the individual [2].

There is emerging evidence to suggest that tattooing may have a positive effect on the mental health of the individual and aid in recovery, along with enabling positive body image and empowerment [8]. Information should be offered to patients with tattoos and those planning to get them regarding the risk of skin disorders, including cancers, being masked by the ink and delayed diagnosis, and vigilance is advised in this regard [9]. 

An assessment of the tattooed areas to be treated to exclude the presence of any underlying lesions is essential prior to treatment. Counselling about the risks and benefits of the treatment and aftercare following tattoo removal should be offered by the appropriate physical health provider. The risks of complications, including the challenges with diagnosing cancerous lesions following tattoo removal, should be discussed with the patient. The aftercare instructions should be shared with the nursing and medical team to allow them to assist with aftercare and monitor for possible complications that can include blistering, bruising, tenderness, itching, and swelling [10].

Mental health professionals should advise caution to patients who are planning to get tattoos about the possible risks associated with them. These can relate to the ink used in tattooing, which has various chemicals; infections [including hepatitis B and C, HIV, herpes, and bacterial infections]; allergic reactions; hypersensitivity; and immunological reactions [11]. For patients who are interested in more information, they should be referred to the healthcare provider who can give them information and guide them to access relevant resources.

Tattoos are acquired by people at various times for differing motivations. This is especially problematic if the tattoos were acquired when the individual was undergoing a mental health crisis or an episode of illness. They can serve as reminders of the past and can act as a hindrance to recovery and rehabilitation. This is especially relevant for facial and other easily visible tattoos that are difficult to conceal. They add to the social stigma and lead to the stereotyping of the individual. Similarly, some tattoos may be linked to gang/group affiliation and can be problematic for the individual who wants to leave that lifestyle. The tattoos that are related to gang/group affiliation can pose a safety hazard for the person in carceral settings and the community.

Mental health professionals need to be mindful of exploring the significance of tattoos with patients where relevant. This can provide an insight into their concerns, which may not always be voiced openly. Professionals should engage in advocacy on behalf of such patients who lack the means to do this for themselves. The improved recovery and rehabilitation will assist with recovery and reduction in risk and recidivism.

Many patients with chronic and severe mental health problems may lack the funds to pay for tattoo removal, given the expense involved. Exploring options for tattoo removal and accessing financial support remains important, especially in patients within forensic mental health and long-stay psychiatric settings. This can be through various sources of funding, including charitable organizations, mental health charities, and patient support organizations. Hospitals should actively support patients’ wishes to have tattoo removal by connecting them with relevant organizations and supporting their transport to the clinic for this, as needed. Clinics that offer tattoo removal should be lobbied to consider offering affordable treatment to patients suffering significant mental health challenges. These could take the form of discounts, extended payment schemes, or connecting them with alternative sources of funding. Similarly, health insurance providers, whether state-funded or private, should be encouraged to support these patients, as it will aid their recovery in the long run and reduce the risk of relapse and recidivism.

## Conclusions

Exploring the personal significance and impact of tattoos is important in mental health settings, especially if they are distressing to the individual. It can allow for a greater understanding of the patient’s cultural and personal belief system. Where appropriate, tattoo removal may assist with the rehabilitation of mental health patients, especially those in forensic mental health settings. Mental health professionals should advocate on behalf of their patients to support them in accessing such services by connecting them with agencies that may be able to provide financial and practical support. Patients within forensic mental health settings may have additional restrictions on their movement and should be supported by providing the necessary transport for treatment.

## References

[1] Ali AY (2018). Tattoos as a window to the mind: is the “body graffiti” skin deep or deeper?. Int J Emerg Ment Health.

[2] Owen DC, Armstrong ML, Koch JR, Roberts AE (2013). College students with body art: well-being or high-risk behavior?. J Psychosoc Nurs Ment Health Serv.

[3] Friedman R, Antoine D, Talamo T (2018). Natural mummies from predynastic Egypt reveal the world's earliest figural tattoos. J Archaeol Sci.

[4] Wohlrab S, Stahl J, Kappeler PM (2007). Modifying the body: motivations for getting tattooed and pierced. Body Image.

[5] Phelan MP, Hunt SA (1998). Prison gang members’ tattoos as identity work: the visual communication of moral careers. Symb Interact.

[6] Palermo GB (2004). Tattooing and tattooed criminals. J Forensic Psychol Pract.

[7] Morlock R, Morlock A (2023). Think before you ink: perception, prevalence, and correlates of tattooing and tattoo regret in US adults. Cureus.

[8] Silverstein S, Santibañez T (2025). A qualitative investigation of tattooing as an adaptive appearance investment: positive body image and eating disorder recovery in a predominantly transgender and gender expansive sample. Eat Disord.

[9] Lebhar J, Jacobs J, Rundle C, Kaplan SJ, Mosca PJ (2024). Skin cancers arising within tattoos: a systematic review. JAAD Int.

[10] Khunger N, Molpariya A, Khunger A (2015). Complications of tattoos and tattoo removal: stop and think before you ink. J Cutan Aesthet Surg.

[11] Petrochko JM, Krakowski AC, Donnelly C, Wilson J, Irick JB, Stawicki S (2019). Tattoo-associated complications and related topics: a comprehensive review. Int J Acad Med.

